# The Leaf Aqueous and Ethanolic Extracts of *Tricalysia okelensis* Hiern. (Rubiaceae) Reverse L‐NAME‐Induced Hypertension, Cardiac, and Vascular Remodeling in Rats

**DOI:** 10.1155/adpp/8990646

**Published:** 2026-09-07

**Authors:** Florence Nokam, Ariane Falone Goumtsa, Sahar Mofidi Tabatabaei, Cédric Wamba Koho, Cherif Mouhamed Moustapha Dial, Kevin Joseph Tidgewell, Pierre Watcho, Elvine Pami Nguelefack-Mbuyo

**Affiliations:** ^1^ Department of Animal Biology, Faculty of Science, Research Unit of Animal Physiology and Phytopharmacology, University of Dschang, P.O. Box 67, Dschang, Cameroon, univ-dschang.org; ^2^ Department of Pharmaceutical Sciences, University of Kentucky, Lexington Kentucky, 40536, USA, uky.edu; ^3^ Faculty of Medicine, Pharmacy and Odonto-Stomatology, Cheikh Anta Diop University, Dakar, Senegal, ucad.sn

**Keywords:** anti-remodeling effect, arterial hypertension, L-NAME, metabolites, molecular networking, *Tricalysia okelensis.*

## Abstract

Arterial hypertension is a worldwide challenging medical condition that continues to raise havoc despite existing treatments, prompting the need to develop new medicine. In a pilot study, we demonstrated that *Tricalysia okelensis* exhibits a vasorelaxant effect. This study evaluates the antihypertensive effect of the leaf aqueous (AETO) and ethanolic (EETO) extracts of *T. okelensis* against L‐NAME‐induced‐hypertension, cardiac, and vascular remodeling. AETO and EETO underwent LC‐MS/MS to identify potential metabolites responsible for the activity. Adult male Wistar rats were orally administered L‐NAME (30 mg/kg/day) for 3 consecutive weeks. L‐NAME administration was continued either alone or with captopril (20 mg/kg/day, p.o.), AETO, or EETO at doses of 85 and 170 mg/kg/day for another 3 consecutive weeks, while the naive group received the vehicle for 6 weeks. Blood pressure (BP) and heart rate were recorded before and weekly after the beginning of each treatment. Plasma angiotensin II (Ang II), serum nitric oxide (NO), lactate dehydrogenase (LDH), cardiac myeloperoxidase (MPO), and NO were assessed at the end of the experiment. Sections from the heart and aorta were analyzed for histological changes and immunohistochemical expression of atrial natriuretic peptide (ANP), endothelial (eNOS), and inducible (iNOS) nitric oxide synthases. AETO and EETO are rich in flavonoids and phenolic compounds. LC‐MS/MS‐based molecular networking enabled the putative annotation of several polyphenolic metabolites, including chlorogenic acid, neochlorogenic acid, rutin, quercetin derivatives, kaempferol derivatives, isorhamnetin, avicularin, and guaijaverin. L‐NAME significantly increased BP and heart rate, Ang II, LDH, cardiac MPO, and heart weight (*p* < 0.05 − 0.001) while decreasing NO (*p* > 0.05). AETO and EETO reversed these alterations and also inhibited L‐NAME‐induced immune cell infiltration, fibrosis, cardiac and vascular eNOS downregulation, and iNOS overexpression, as well as ANP upregulation in heart tissue. AETO and EETO exhibit antihypertensive and anti‐remodeling effects probably by modulating the renin‐angiotensin system, improving endothelial function, and reducing inflammation and fibrosis.

## 1. Introduction

Systemic persistent high blood pressure, known as arterial hypertension (AHT), is a very prevalent noncommunicable chronic disease that globally affects 32% of adults aged 30–79 years in high‐income countries versus 34% in low‐income countries [1]. It is a very challenging health condition associated with a high risk of developing target organ damage, including the heart, kidney, vessel, brain, and eye [2]. AHT is a life‐threatening medical affliction accompanied by increased mortality from cardiovascular disease and kidney disease [1]. It is responsible for 13% of premature deaths [3].

Endothelial dysfunction is recognized as the hallmark of primary hypertension, which accounts for 85%–95% of all cases of AHT [4–6]. Endothelial dysfunction originates from an imbalance between relaxing and contracting factors in favor of the latter [7, 8]. Its underlying mechanisms include a reduction in endothelial nitric oxide (NO), the foremost endothelial relaxing factor synthesized by nitric oxide synthase (eNOS), which plays a key role in blood pressure regulation [7], and the increase in vascular contracting agents. Besides, the stimulation of the renin‐angiotensin‐aldosterone system (RAAS), culminating in the release of tremendous amounts of angiotensin II (Ang II), is another pathway responsible for the occurrence of AHT [9–11]. Moreover, Ang II activates inflammatory and fibrotic pathways leading to vascular and cardiac remodeling that further sustains AHT. One of the well‐established experimental animal models that closely recapitulates these human pathophysiological features of primary AHT and the associated target organs’ damages is the L‐NAME‐induced AHT [12].

The effective control of AHT is the ideal solution to prevent cardiovascular incidents. Unfortunately, high blood pressure control remains largely unmet in low‐and‐middle‐income countries, where only 10.3% of patients achieve effective blood pressure control [13]. In the case of sub‐Saharan Africa, reasons for uncontrolled hypertension include therapy failure due to resistance to the available medications and low socioeconomic status [14, 15]. In addition to this, antihypertensive drugs have many side effects that limit patient adherence to the therapeutic regimen, justifying their reliance on alternative solutions, including plant‐based treatment.

Medicinal plants have outstandingly contributed to the development and expansion of the pharmaceutical industries. As such, they represent valuable tools for the discovery and development of new, effective, and well‐tolerated antihypertensive medications. Plants from the Rubiaceae family are known to exhibit potent cardiovascular activities, including against AHT, myocardial infarction, heart failure, and Ang II‐induced myocardial fibrosis [16–21]. *Tricalysia okelensis* is a plant of the Rubiaceae family found in subtropical and tropical regions in Africa and Asia [22]. It is traditionally used in the western region of Cameroon to treat diarrhea [23]. Preliminary qualitative phytochemical analysis of ethanol/water extracts of *T. okelensis* performed by Rene et al. [23] revealed the presence of saponins, phenolic compounds, coumarins, reduced sugar, polysaccharides, triterpenes, tannins, alkaloids, and volatile oils. Two ent‐kaurane glycosides, namely, tricalysioside V and tricalysioside W, were isolated from the roots of *T. okelensis* by Xu et al. [22]. A pharmacological study by Rene et al. [23] showed that *T. okelensis* possesses antimicrobial and antidiarrheal effects.

In a pilot study, we found that the aqueous extract from the leaves of *T*. *okelensis* exhibits a vasorelaxant effect. It was then hypothesized that this plant may reduce AHT and related organ damage through the improvement of NO bioavailability and the inhibition of the renin‐angiotensin system. This study was therefore undertaken to evaluate the blood pressure‐lowering and the target‐organ protective effects of the aqueous and ethanolic extracts from the leaves of *T. okelensis* against N^ω^‐nitro‐L‐arginine methyl ester (L‐NAME)‐induced hypertension.

## 2. Materials and Methods

### 2.1. Reagents

L‐NAME, sodium pyruvate, and hexadecyltrimethylammonium were purchased from Sigma‐Aldrich (Germany). eNOS and iNOS polyclonal antibodies, angiotensin 2 ELISA kit, and poly‐HRP antirabbit detection kit were all bought from Elabscience (USA). DAB was obtained from Quartett (Berlin, Germany). The ANP antibody was purchased from EMD Millipore Corporation (USA). Captopril was obtained from Denk Pharma (Germany). NaCl and NADH were purchased from Carl‐Roth (Kalshur, Germany).

### 2.2. Animals

Male adult Wistar rats, aged 3‐4 months with body weights ranging from 200 to 280 g, were used. The animals were reared in the animal facility of the Research Unit of Animal Physiology and Phytopharmacology at the University of Dschang (Cameroon). They were housed in plastic cages under a natural 12 h light/12 h dark cycle at a temperature of 22°C ± 2°C. Animals were provided with food and water *ad libitum*. The study was approved by the internal committee of the Department of Animal Biology of the University of Dschang (16‐25/FS‐CERFAS), which ensures that all animal procedures are conducted in accordance with the ethical standards for animal use and care of the European Parliament’s 2010/63/EU law.

### 2.3. Plant Collection and Extraction

The fresh leaves of *T. okelensis* were collected in Fongo‐Ndeng, a locality of the Dschang Subdivision (West Region, Cameroon). A specimen of this plant was authenticated at the National Herbarium of Cameroon (HNC) by comparison with an existing voucher specimen N°35629/HNC. The leaves were washed, shade‐dried, and ground. The powder obtained was used to prepare the aqueous and ethanolic extracts.

To prepare the aqueous extract (AETO), 400 g of the leaf powder of *T. okelensis* was soaked in 4 L of distilled water, boiled, and filtered with Whatman No. 2 paper. After filtration, the residue was again extracted and filtered as previously mentioned. The two filtrates were mixed and concentrated in a ventilated oven at 40°C until dryness. The resulting extract (45.26 g) was kept in a closed container at 4°C.

The ethanolic extract of *T. okelensis* (EETO) was prepared by macerating 400 g of leaf powder in 4 L ethanol for 48 h at room temperature with occasional stirring. The maceration was filtered using Whatman No. 2 paper. The residue obtained was once more macerated in 2 L ethanol for 24 h and filtered. All filtrates were pooled and concentrated at 79°C using a rotary evaporator. Excess solvent was removed by putting the extract in a ventilated oven at 40°C until complete evaporation. The ethanolic extract obtained (35.59 g) was stored in a closed container at 4°C.

### 2.4. LC‐MS/MS‐Based Metabolomics Analysis of Aqueous and Ethanolic Extracts of *Tricalysia okelensis*

LC‐MS/MS‐based molecular networking was performed using the GNPS2 platform. Chromatographic separation was carried out on a Shimadzu DGU‐20A 3R system coupled to a SCIEX TripleTOF 5600 mass spectrometer equipped with a DuoSpray electrospray ionization (ESI) source. MS data were acquired in both positive and negative ionization modes over an m/z range of 100–1200 using a data‐dependent acquisition method enabling simultaneous collection of MS and MS/MS spectra. The extracts were prepared in methanol/water (50/50) at a concentration of 3 mg/mL, and 30 μL was injected for each LC‐MS/MS run. Molecular networking and data visualization were conducted using the online GNPS2 workflow [24] and spectral libraries available through the GNPS2 platform (https://gnps2.org). A summary of the spectral data is provided in the supporting information, and all raw data and molecular networking jobs are publicly accessible on GNPS2 via the associated task ID numbers.

### 2.5. Evaluation of the Antihypertensive Activity of the Aqueous and Ethanolic Extracts From the Leaves of *Tricalysia okelensis*

#### 2.5.1. Dose Calculation

The doses of extracts used in this study were calculated using the half maximal effective concentration (EC_50_) and the circulating blood volume. The rat blood volume was determined using the following formula from [25]: blood volume = 0.06 × BW + 0.77. Briefly, the EC_50_ of the aqueous extract was found to be 130 μg/mL according to a pilot study evidencing the vasorelaxant effect of this extract (Supporting Figure S2). The EC_50_ value was multiplied by the circulating blood volume, which was found to be 12.77 mL for a rat with an average weight of 200 g. This calculation yielded a result of 1660.1 μg. According to Liu et al. [26], only 5%–10% polyphenols are absorbed in the gastro‐intestinal tract. Given the richness of *T. okelensis* extracts in polyphenols (Supporting Table S1), we considered that 1660.1 μg obtained represents 5% of the extract absorbed. To determine the required dose of extract to achieve at least the EC_50_ value obtained in vitro, 1660.1 μg was multiplied by 100 and then divided by 5 to obtain the dose necessary for complete absorption. The dose obtained was 33,202 μg/200 g, which is also equal to 166.01 mg/kg ≈ 170 mg/kg/day, and the other dose was obtained by dividing 170 by 2.

#### 2.5.2. Induction of Arterial Hypertension and Grouping

AHT was induced by chronic administration (6 consecutive weeks) of L‐NAME dissolved in distilled water, at the dose of 30 mg/kg once a day as previously described [27]. Before starting the experiment, animals were acclimatized to the indirect blood pressure recording for 3 weeks, after which the baseline value was measured. Rats were then assigned into 2 batches based on the baseline blood pressure value. With the exception of batch 1 (naive; *n* = 9), which received the vehicle (distilled water) throughout the experiment, all the rats of batch 2 received L‐NAME for 3 consecutive weeks. At the end of the third week, the animals of batch 2 with a blood pressure above 140/90 mm Hg were randomly assigned into 6 groups of 9 rats each as follows:

- Group 2, disease control rats that received L‐NAME
- Group 3 received L‐NAME + captopril (20 mg/kg/day) [27].
- Group 4 was treated with L‐NAME + AETO at the dose of 85 mg/kg/day
- Group 5 received L‐NAME + AETO at the dose of 170 mg/kg/day
- Group 6 was treated with L‐NAME + EETO at the dose of 85 mg/kg/day
- Group 7 received L‐NAME + EETO at the dose of 170 mg/kg/day

All substances were administered orally by gavage at a volume of 1 mL/100 g body weight. Blood pressure and heart rate were indirectly recorded at baseline and at the end of each week using a noninvasive tail cuff plethysmograph (IITC Life Science MRBP). At the end of the experiment, animals were anesthetized by intraperitoneal injection of sodium thiopental at the dose of 50 mg/kg, and blood was collected from the abdominal artery into EDTA and EDTA‐free tubes. After a 15 min centrifugation at 3500 rpm at 4°C, plasma and serum were collected and used for the assessment of Ang II and lactate dehydrogenase (LDH), respectively. The thoracic aorta and the heart were collected, washed in isotonic saline solution, weighed, and further used for biochemical assessment or for morphometric, histopathologic, and immunohistochemistry analysis.

#### 2.5.3. Biochemical Assessment

The determination of LDH activity was performed using a modified method of “la Société Française de Biologie Clinique” as previously described [28]. Changes in absorbance following NADH oxidation were measured 30 sec after the initiation of the reaction and 1, 2, and 3 min later at 340 nm using a microplate reader (Multiskan FC, Thermo Fisher). The following formula was used to determine LDH activity:

(1) LDHU/L=ΔDOmin×4921.

The plasma Ang II level was determined using a commercial ELISA kit according to the manufacturer’s instructions (Elabscience; Cat. # E‐EL‐R1430, Lot: V3qxHacD8P). Serum and cardiac NO were quantified spectrophotometrically as previously described [29]. MPO was determined in cardiac and aorta homogenates as previously reported [28]. The change in optical density was monitored at 450 nm.

#### 2.5.4. Morphometric and Histopathological Analysis

Morphometric and histopathological analysis were performed in a blinded manner. The investigators responsible for mounting or analyzing the histological slides remained unaware of the specific treatments involved. The heart and thoracic aorta of three individual rats were harvested and fixed in 10% formaldehyde prepared in NaCl (0.9%) solution. The tissues were embedded into paraffin, and 4 μm sections cut transversally were stained with hematoxylin‐eosin (H&E) or with Masson’s trichrome. Three sections per organ per rat were photographed and served for morphometric analysis using a light microscope (LEICA DM750) coupled to a camera (LEICA ICC50‐E). Data from each rat were averaged and served as a single value [30]. The following parameters were quantified by means of Image J. software: the total heart surface, left ventricular (LV) wall thickness, LV lumen surface, LV wall to LV lumen ratio, aortic wall thickness, aortic lumen surface, media thickness, and media to lumen ratio.

#### 2.5.5. Immunohistochemistry Analysis

Immunohistochemistry analysis of endothelial and inducible eNOS and iNOS and atrial natriuretic peptide (ANP) was performed on 4 μm paraffin‐embedded sections of the left ventricle or thoracic aorta mounted on ionized superfrost slides. After dewaxing, the sections were hydrated, and the antigen was retrieved for 30 min in Tris‐EDTA buffer pH = 9. The sections were treated with 0.3% hydrogen peroxide for 5 min to inhibit endogenous peroxidase activity and washed with Tris‐HCl buffer pH = 7.4 [31]. Sections were then immunolabeled using a polyclonal antibody for eNOS (cat. # E‐AB‐32267; dilution 1:100; Elabscience), a polyclonal antibody for iNOS (cat. # E‐AB‐70051; dilution 1:300; Elabscience), and rabbit antibody ANP (EMD Millipore Corporation; dilution 1:1000) according to the manufacturer’s instructions. The expression of eNOS, iNOS, and ANP was estimated using Image J software. To avoid subjectivity that could bias the readings, samples were codified, and the analyses were performed by an experimenter who was blinded to the procedure.

### 2.6. Statistical Analysis

All the results are expressed as mean ± SEM. The statistical analysis was performed using GraphPad Prism 8.4.3 software. Data on LDH, Ang II, heart, left ventricle, and aorta weight, cardiac and aorta morphometry, and NO and MPO assessments were analyzed using one‐way analysis of variance (ANOVA) followed by Tukey’s post hoc test. To analyze longitudinal data (blood pressure, heart rate, and body weight), a two‐way ANOVA with repeated measures followed by Tukey’s post hoc test was used. But prior to this, the Maulchy test was performed to assess the sphericity and the Geisser‐Greenhouse correction was applied when necessary. Significant differences between means were set at *p* < 0.05.

## 3. Results

### 3.1. Phytochemical Composition of the Aqueous and Ethanolic Extracts of *Tricalysia okelensis*

**FIGURE 1:**
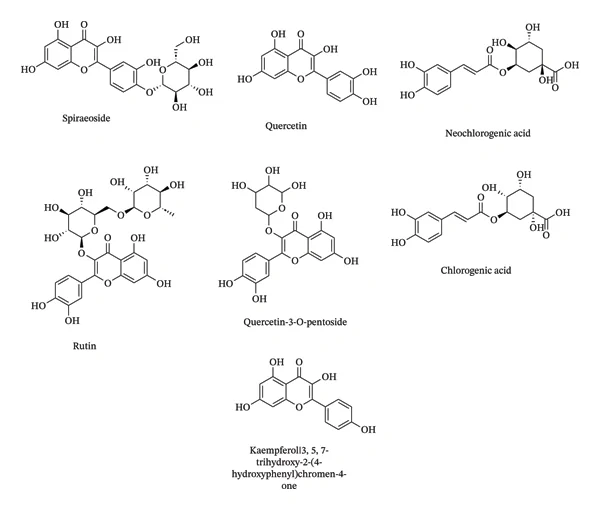
Putatively annotated compounds in the aqueous extract of *Tricalysia okelensis* through LC‐MS/MS molecular networking.

**FIGURE 2:**
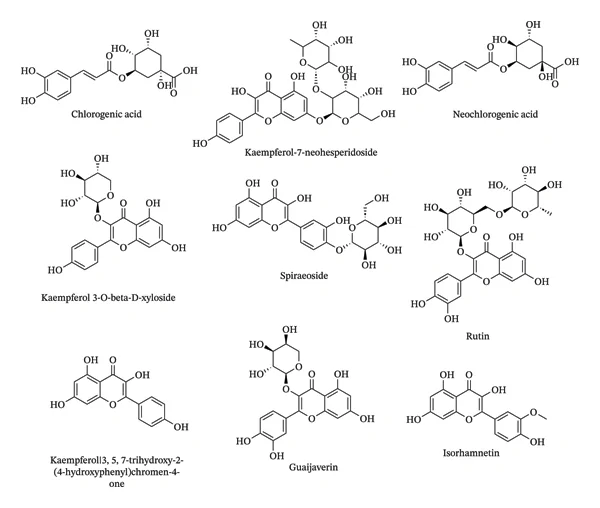
Putatively annotated compounds in the ethanolic extract of *Tricalysia okelensis* through LC‐MS/MS molecular networking.

LC‐MS/MS‐based molecular networking was performed in both positive and negative ionization modes to comprehensively characterize the phytochemical composition of the aqueous and ethanolic extracts. Molecular networking analysis revealed chlorogenic acid and neochlorogenic acid, two positional isomers belonging to the phenolic acid class, as major metabolites present in both extracts, together with rutin, a flavonoid glycoside, and several additional polyphenolic constituents. Overall, dereplication analysis enabled the putative annotation of 7 compounds in the aqueous extract and 9 compounds in the ethanolic extract (Figures 1 and 2). LC‐MS traces (Figures S18 and S19) showed two major ions at m/z 353 [M − H]^−^ in negative mode and m/z 355 [M + H]^+^ in positive mode, which were annotated through GNPS2 molecular networking and spectral library matching as chlorogenic acid and its isomer neochlorogenic acid. Another prominent ion detected at m/z 609 [M − H]^−^ and m/z 611 [M + H]^+^ was identified as rutin. The aqueous extract was primarily characterized by chlorogenic acid derivatives, rutin, quercetin, quercetin‐3‐O‐pentoside, spiraeoside, and kaempferol derivatives, whereas the ethanolic extract exhibited a chemically more diverse flavonoid profile containing chlorogenic acid, neochlorogenic acid, rutin, spiraeoside, kaempferol derivatives, kaempferol‐3‐O‐β‐D‐xyloside, kaempferol‐7‐neohesperidoside, and isorhamnetin. In addition, an ion detected at m/z 433 [M − H]^−^ in the ethanolic extract as another major compound (Figure S19) was tentatively annotated as guaijaverin based on molecular networking analysis (Figure S20). Additional molecular networking data, dereplication workflow, and annotated spectra are provided in the Supporting Information (Figures S3–S17).

### 3.2. Effect of *T. okelensis* Extracts on Hemodynamic Parameters

**FIGURE 3:**
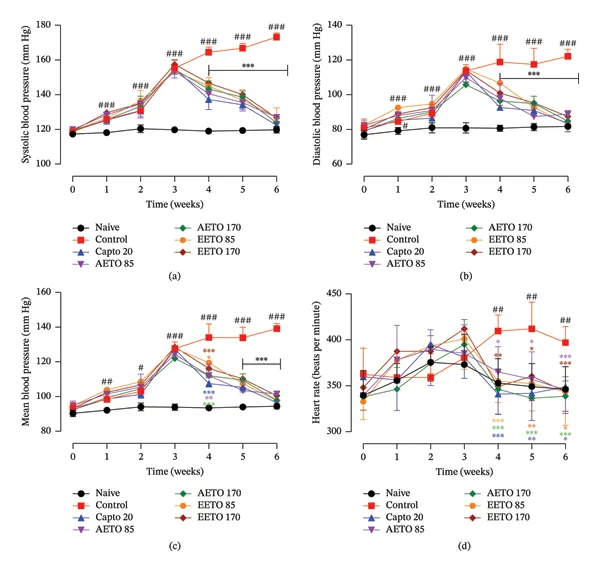
The effects of *Tricalysia okelensis* administration on hemodynamic parameters show blood pressure‐lowering effect (a, b, and c) and heart rate‐lowering effect (d). Data are expressed as mean ± SEM (*n* = 8‐9). Data were analyzed using two‐way repeated‐measures ANOVA followed by Tukey’s post hoc test. ^#^ *p* < 0.05, ^##^ *p* < 0.01, ^###^ *p* < 0.001 significantly different compared to the naive. ^∗∗^ *p* < 0.01, ^∗∗∗^ *p* < 0.001 significantly different compared to the control group. Capto 20 = captopril (20 mg/kg); AETO 85 and AETO 170 = aqueous extracts of *Tricalysia okelensis* at doses of 85 and 170 mg/kg, respectively; EETO 85 and EETO 170 = ethanolic extracts of *Tricalysia okelensis* at doses of 85 and 170 mg/kg, respectively.

Chronic administration of L‐NAME alone progressively and significantly increased systolic (SBP), diastolic (DBP), and mean (MBP) blood pressure (*p* < 0.001) from the first week until the end of the experiment. At week 6, SBP in the control group reached 173.11 ± 8.38 mmHg versus 119.77 ± 0.39 mmHg in the naive group (Figure 3a). DBP increased from 81.66 ± 0.63 mmHg in the naive group up to 122.11 ± 6.81 mmHg in rats treated with L‐NAME alone (Figure 3b), while MBP in the control group was 139.10 ± 0.97 mmHg vs. 94.37 ± 0.57 mmHg in the naïve group (Figure 3c). The administration of both extracts of *T. okelensis* at all doses along with captopril, during the last 3 weeks, markedly attenuated L‐NAME‐evoked hypertension (*p* < 0.001). One rat in the captopril group died a day before treatment with captopril started.

As depicted in Figure 3c, it can be observed that, during the first 3 weeks, the administration of L‐NAME alone caused no significant variation in heart rate (*p* > 0.05). However, from week 4 to week 6, the heart rate of rats of the control group significantly rose (*p* < 0.001) as a result of L‐NAME treatment. The change in heart rate was 397 ± 5.77 bpm in the control group compared to 346.00 ± 8.26 bpm in the naïve group at the end of the experiment. The administration of the plant extracts or captopril totally abolished this effect (*p* < 0.001).

### 3.3. Effects of the Leaf Aqueous and Ethanolic Extracts of *T. okelensis* on Biochemical Parameters

#### 3.3.1. Effects on Plasma Ang II

**FIGURE 4:**
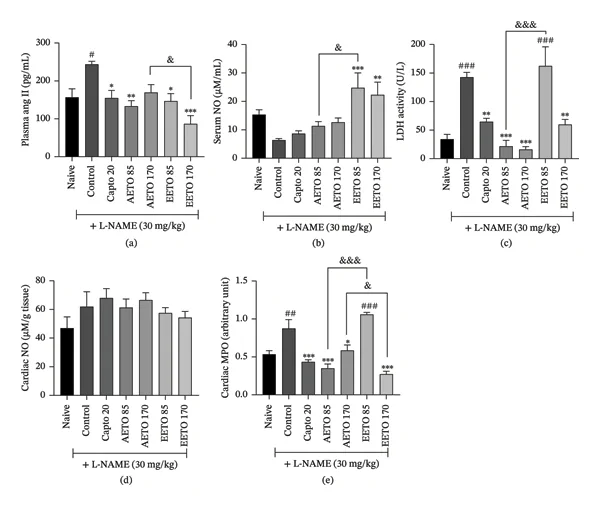
Effect of the leaf aqueous and ethanolic extracts of *Tricalysia okelensis* on plasma angiotensin II level (a), serum nitric oxide (b), LDH activity (c), cardiac nitric oxide (d), and MPO (e) in L‐NAME‐induced hypertensive rats. Data are expressed as mean ± SEM ((*n* = 7‐8) for serum parameters and *n* = 5 for tissue parameters). Data were analyzed using one‐way ANOVA with Tukey’s test. ^#^ *p* < 0.05, ^###^ *p* < 0.001 significantly different compared to the naive group; ^∗^ *p* < 0.05, ^∗∗^ *p* < 0.01, ^∗∗∗^ *p* < 0.001 compared to the control group. ^&^ *p* < 0.05, ^&&&^ *p* < 0.001 significant difference between plant extracts. Capto 20 = captopril (20 mg/kg); AETO 85 and AETO 170 = aqueous extracts of *Tricalysia okelensis* at doses of 85 and 170 mg/kg, respectively; EETO 85 and EETO 170 = ethanolic extracts of *Tricalysia okelensis* at doses of 85 and 170 mg/kg, respectively.

Plasma Ang II levels increased by 55.64% in animals that received only L‐NAME (*p* < 0.05; *R* ^2^ = 0.47; 95% IC: −169.70 to −4.54) compared to the naive group (Figure 4a). Treatment with the plant extracts similar to captopril significantly reduced the amount of circulating Ang II (*p* < 0.05, *p* < 0.01, and *p* < 0.001), with the ethanolic extract at the dose of 170 mg/kg being the most active (*p* < 0.001; *R* ^2^ = 0.47; IC: 74.51–239.60). Nevertheless, no significant difference (*p* > 0.05) between the two extracts and captopril was observed while the effect of the aqueous extract at the dose of 170 mg/kg was statistically different from that of the ethanolic extract at the same dose (*p* < 0.05). Globally, the administration of the plant extracts lowered the Ang II level to a value similar to that of the naive group.

#### 3.3.2. Effects on Serum NO

As observed in Figure 4b, L‐NAME administration alone caused a drastic depletion in serum NO levels compared to the naive group. The amount of NO in the serum of animals of the naive group was 2.41 times that of animals receiving L‐NAME only. However, this effect was not statistically significant (*p* > 0.05; *R* ^2^ = 0.41; 95% CI: −3.51–31.47) despite this drastic drop. Treatment with the plant extracts, especially the ethanolic extract, significantly increased the serum NO concentration (*p* < 0.001 and *p* < 0.01, respectively, for the doses 85 mg/g and 170 mg/kg) while captopril failed to reverse the drop in NO elicited by L‐NAME. Moreover, a significant (*p* < 0.05) difference between the aqueous and ethanolic extracts at the dose of 85 mg/kg has been observed with the ethanolic extract being the most active on this parameter (Figure 4b).

#### 3.3.3. Effect of the Aqueous and Ethanolic Extracts of *Tricalysia okelensis* on LDH Activity

When compared to the naive group, there was a surge in LDH levels (*p* < 0.001; *R* ^2^ = 0.69; 95% IC: −173.60 to −43.55) in rats of the control group as a result of chronic L‐NAME administration (Figure 4c). This increase in serum LDH was successfully reversed by the administration of captopril or the plant extracts. The leaf aqueous extract of *T. okelensis* at all doses exhibited the most powerful effect compared to captopril (reference drug) or the ethanolic extract, which was only active at the dose of 170 mg/kg.

#### 3.3.4. Effects of *Tricalysia okelensis* Leaves on Cardiac Nitric Oxide

The results depicted in Figure 4d show that no significant (*p* > 0.05) change in cardiac NO was observed among the experimental groups.

#### 3.3.5. Effects of *Tricalysia okelensis* Leaves on Cardiac Myeloperoxidase

A 64% increase in cardiac MPO levels was observed in animals that received L‐NAME alone compared to the naive group (*p* < 0.01; *R* ^2^ = 0.81; 95% CI: −0.62 to −006). This parameter was significantly (*p* < 0.05 and *p* < 0.001) reduced when *T. okelensis* extracts or captopril were administered. It is worth noticing that the aqueous extract reduced cardiac MPO more effectively than the ethanolic extract. MPO levels were decreased by both doses of the aqueous extract used, although the largest reduction (*p* < 0.001; *R* ^2^ = 0.81; 95% CI: 0.24–0.81) was seen at the dose of 85 mg/kg, whereas this decrease was only observed at the dose of 170 of the ethanolic extract.

### 3.4. Effect of the Aqueous and Ethanolic Extracts From *Tricalysia okelensis* on Body Weight and Organ Mass

At the end of the experiment, the body weight gain was slowed down in L‐NAME‐treated rats (*p* > 0.05; *R* ^2^ = 0.25; 95% CI: −19.62–48.06). This effect was potentiated by *T. okelensis* extracts at the dose of 170 mg/kg (*p* < 0.05; *R* ^2^ = 0.25; 95% CI: 5.82–73.51) and to a lesser extent by captopril (Supporting Table S2).

Following chronic administration of L‐NAME alone, a significant increase in the total heart weight (*p* < 0.001; *R* ^2^ = 0.49; 95% CI: −70.99 to −16.37) was observed when compared to the naive group. This increase in heart weight elicited by L‐NAME was reversed by the treatment with both plant extracts and captopril (*p* < 0.01, *p* < 0.001).

Although not statistically significant (*p* > 0.05; *R* ^2^ = 0.40; 95% CI: −50.12 to 1.42), L‐NAME induced a 17.44% increase in left ventricle mass. Only captopril and the aqueous extract at the dose of 170 mg/kg significantly (*p* < 0.05 and *p* < 0.01) counteract the effect of L‐NAME.

Apart from the rats treated with L‐NAME and the ethanolic extract of *T. okelensis* at the dose of 170 mg/kg in which a decrease in aorta mass was observed (*p* < 0.01; *R* ^2^ = 0.24; 95% CI: 0.25–19.01), no significant change in aorta mass was noticed in other experimental groups (Supporting Table S2).

### 3.5. Effect of the Aqueous and Ethanolic Extracts of *Tricalysia okelensis* on Cardiac Morphometry and Histology

**FIGURE 5:**
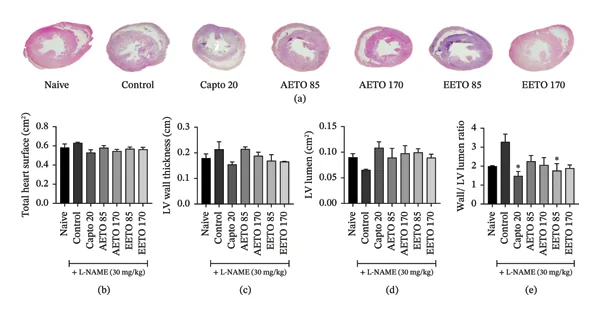
Hematoxylin and eosin‐stained sections of the heart showing the effects of *Tricalysia okelensis* extracts on cardiac morphometry (a) and histomorphometric analysis (b to e). The magnification power is 1.5 under the binocular magnifying glass. Data are expressed as mean ± SEM. (*n* = 3) and were analyzed using one‐way ANOVA with Tukey’s test. ^∗^ *p* < 0.05 significant difference compared to the control group. Capto 20 = captopril (20 mg/kg); AETO 85 and AETO 170 = aqueous extracts of *Tricalysia okelensis* at the doses of 85, 170, respectively; and EETO 85 and EETO 170 = ethanolic extracts of *Tricalysia okelensis* at the doses of 85 and 170, respectively.

Histomorphometric analysis of heart sections stained with hematoxylin and eosin shows that L‐NAME administration caused cardiac remodeling. The total heart surface did not differ significantly among the different experimental groups (*p* > 0.05; *R* ^2^ = 0.41) (Figure 5b). In comparison to the naive group, rats in the control group presented a slight enlargement of the LV wall chamber that was associated with a 27.51% reduction in the LV lumen and a 64.46% nonsignificant increase (*p* > 0.05; 95% IC: −0.17 to 0.07) in the LV wall to lumen ratio (Figure 5c). These changes in cardiac morphometry were markedly reversed by the administration of captopril, especially the LV lumen and the wall to lumen ratio (*p* < 0.05 and *p* < 0.001). Globally, treatment with the plant extracts nearly normalized the morphometric changes elicited by L‐NAME, except the wall to lumen ratio, which was elevated in animals that received the aqueous extract at the dose of 85 mg/kg. However, it is important to notice that the ethanolic extract, similar to captopril, significantly reduced the LV wall to lumen ratio (*p* < 0.05; 95% CI: 0.02–3.01; Figure 5e).

**FIGURE 6:**
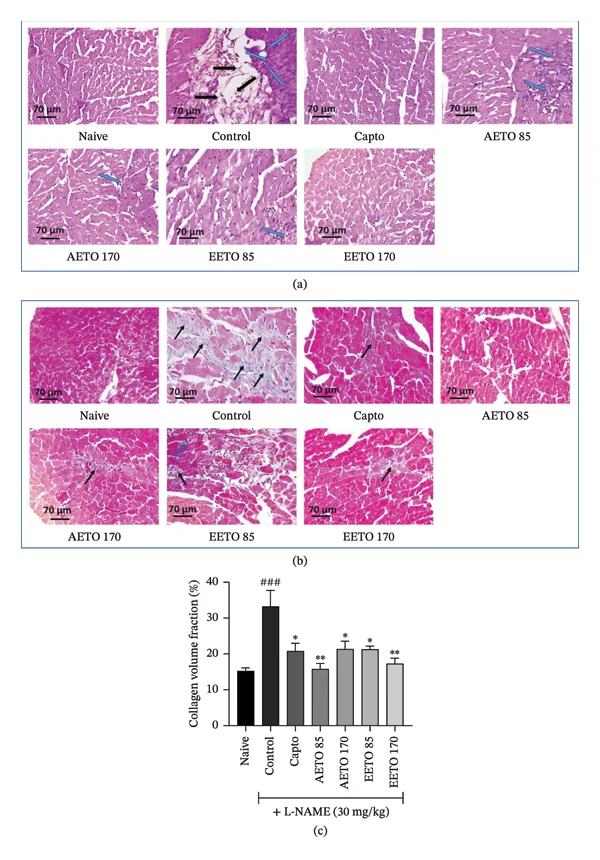
Effects of *Tricalysia okelensis* on histological sections of the heart stained with hematoxylin and eosin (a) or Masson’s trichrome (b and c). Magnification X400. Blue arrows represent inflammation, thick black arrows represent areas of cell death, and thin black arrows illustrate fibrosis. Data are expressed as mean ± SEM (*n* = 3) and were analyzed using one‐way ANOVA with Tukey’s test. ^###^ *p* < 0.001 significantly different compared to the naive group. ^∗^ *p* < 0.05, ^∗∗^ *p* < 0.01 significant difference compared to the control group. AETO 85 and AETO 170: aqueous extracts of *Tricalysia okelensis* at doses of 85 and 170 mg/kg, respectively; EETO 85 and EETO 170: ethanolic extracts of *Tricalysia okelensis* at doses of 85 and 170 mg/kg, respectively; Capto 20 = captopril (20 mg/kg).

Figure 6 shows the effect of *T*. *okelensis* extracts on histological cardiac sections stained with hematoxylin and eosin or Masson’s trichrome. It appears from this figure that animals from the naive group showed a good architecture of the cardiac tissue with a normal arrangement of the muscle fibers compared to the animals receiving L‐NAME administration alone (control group), in whom a significant area of necrosis as well as leukocyte infiltration are observed. In animals treated with captopril, AETO, and EETO, we noticed an improvement in the cardiac histology except for AETO at the dose of 85 mg/kg in which an increase of leukocyte infiltration is still observed.

It is also observed that the long‐term administration of L‐NAME alone (control group) led to an increase in collagen synthesis as revealed by the Masson’s trichrome staining of the cardiac tissue (Figure 6b). In addition to that, the collagen volume fraction (CVF) was significantly higher in the control group compared to the naïve group (*p* < 0.01; *R* ^2^ = 0.75; 95% CI: −28.96 to −7.03). Treatment with either captopril or plant extracts (AETO and EETO) significantly reduced L‐NAME‐induced increases in CVF, as shown in Figure 6c (*p* < 0.05 and *p* < 0.01).

### 3.6. Effect of *Tricalysia okelensis* Leaves on Aorta Morphometry and Histology

The administration of L‐NAME caused a nonsignificant increase in the vascular wall (*p* > 0.05; *R* ^2^ = 0.51; 95% IC: −0.26 to 0.06) and media (*p* > 0.05; *R* ^2^ = 0.50; 95% IC: −0.21 to 0.008) of animals in the control group. Although not statistically significant, this increase in vascular wall and media was associated with a thickening of collagen fibers. After treatment with the plant extracts, especially at the dose of 85 mg/kg, the vascular morphometry and histology were similar to those of the animals in the naive group (Supporting Figure S2).

### 3.7. Immunohistochemical Analysis

#### 3.7.1. Effect of *Tricalysia okelensis* Leaf Extracts on eNOS Expression

**FIGURE 7:**
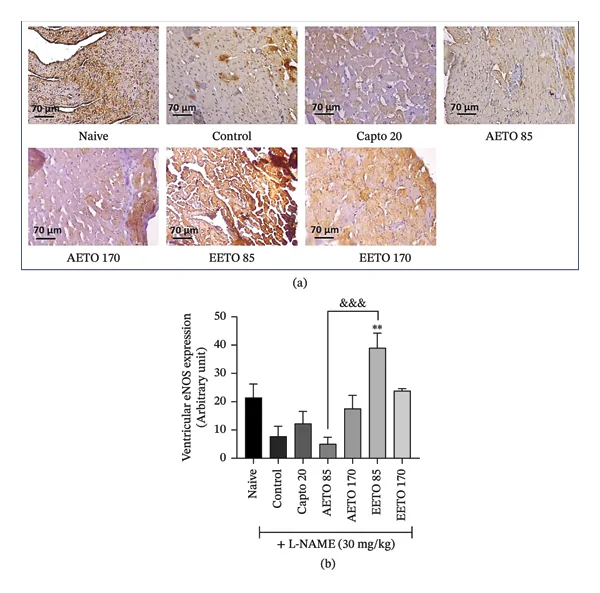
Effects of *Tricalysia okelensis* extracts on cardiac eNOS expression. (a) Immunohistochemical sections. (b) Quantitative graph. Data are expressed as mean ± SEM (*n* = 3) and were analyzed using one‐way ANOVA with Tukey’s test. ^∗∗^ *p* < 0.01 significant difference compared to the control group. ^&&&^ *p* < 0.001 significant difference between the two plant extracts. AETO 85 and AETO 170: aqueous extracts of *Tricalysia okelensis* at doses of 85 and 170 mg/kg respectively; EETO 85 and EETO 170: ethanolic extracts of *Tricalysia okelensis* at doses of 85 and 170 mg/kg, respectively; Capto 20 = captopril (20 mg/kg).

**FIGURE 8:**
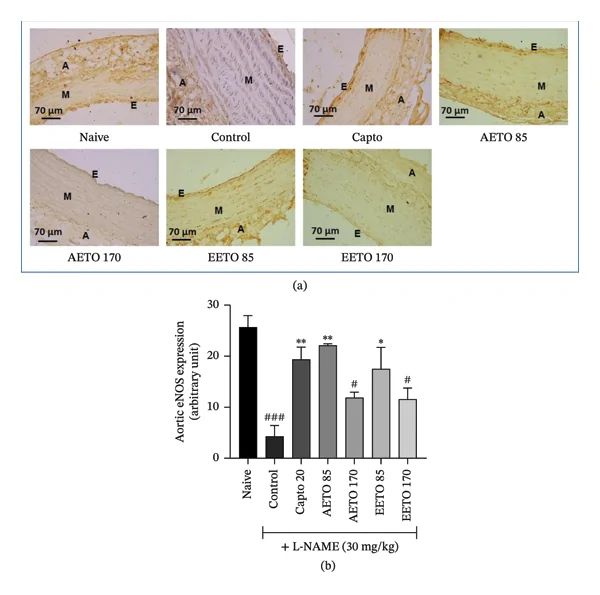
Effects of *Tricalysia okelensis* extracts on aortic eNOS expression. (a) Immunohistochemical sections. (b) Quantitative graph. Data are expressed as mean ± SEM (*n* = 3) and were analyzed using one‐way ANOVA with Tukey’s test. ^#^ *p* < 0.05, ^###^ *p* < 0.001 significantly different compared to the naive. ^∗^ *p* < 0.05, ^∗∗^ *p* < 0.01 significantly different compared to the control group. AETO 85 and AETO 170: aqueous extracts of *Tricalysia okelensis* at doses of 85 and 170 mg/kg respectively; EETO 85 and EETO 170: ethanolic extracts of *Tricalysia okelensis* at doses of 85 and 170 mg/kg, respectively; Capto 20 = captopril (20 mg/kg).

Intense expression of eNOS was noticed in the heart and in the aorta of normotensive rats (naive group). Conversely, chronic administration of L‐NAME caused a drastic decline in eNOS expression. Rats in the control group experienced a 63.84% decrease in eNOS expression (*p* > 0.05; *R* ^2^ = 0.78; 95% CI: −5.61–32.91) in their hearts (Figure 7) and an 83.36% decrease in the same parameter (*p* < 0.001; *R* ^2^ = 0.80; 95% CI: 9.88–32.92) in their aorta (Figure 8) as a result of chronic L‐NAME administration. Captopril was able to reverse L‐NAME‐induced downregulation of eNOS only in the aorta. The cardiac expression of eNOS was similar to that of the naive group following *T. okelensis* administration at the dose of 170 mg/kg, regardless of the extract used. Moreover, eNOS expression was highly improved in the hearts of rats that received the ethanolic extract of *T. okelensis* at the dose of 85 mg/kg. Notably, a significant difference (*p* < 0.001; *R* ^2^ = 0.78; 95% CI: −50.53 to −12.01) was observed between the aqueous and ethanolic extracts at the dose of 85 mg/kg (Figure 7b).

#### 3.7.2. Effect of *Tricalysia okelensis* Leaf Extracts on iNOS Expression

**FIGURE 9:**
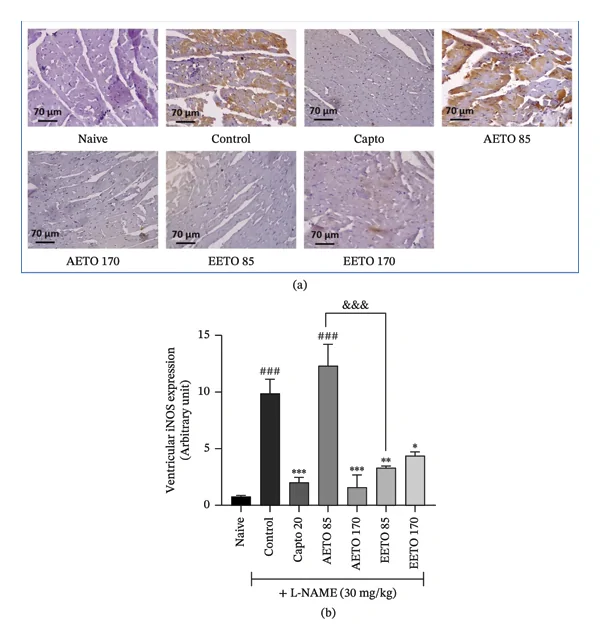
Effects of *Tricalysia okelensis* extracts on cardiac iNOS expression. (a) Immunohistochemical sections. (b) Quantitative graph. Data are expressed as mean ± SEM (*n* = 3) and were analyzed using one‐way ANOVA with Tukey’s test. ^#^^###^ *p* < 0.001 significantly different compared to the naive. ^∗^ *p* < 0.05, ^∗∗^ *p* < 0.01, ^∗∗∗^ *p* < 0.001 significantly different compared to the control group. ^&&&^ *p* < 0.001 significant difference between plant extracts. AETO 85 and AETO 170: aqueous extracts of *Tricalysia okelensis* at doses of 85 and 170 mg/kg, respectively; EETO 85 and EETO 170: ethanolic extracts of *Tricalysia okelensis* at doses of 85 and 170 mg/kg, respectively; Capto 20 = captopril (20 mg/kg).

**FIGURE 10:**
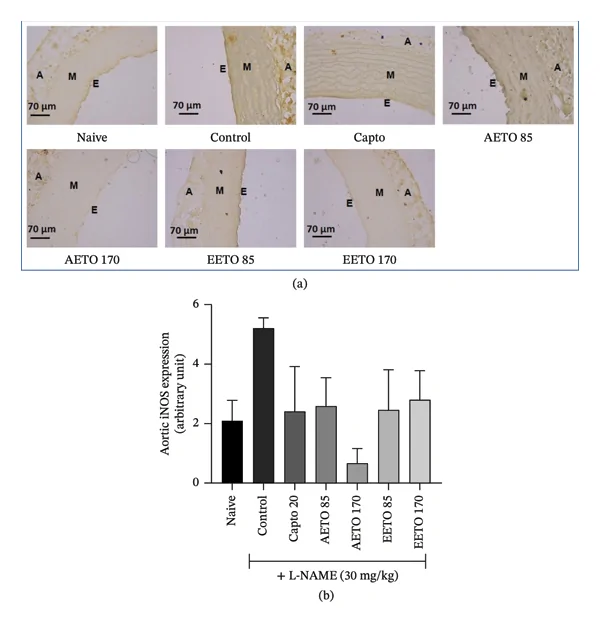
Effects of *Tricalysia okelensis* extracts on aortic iNOS expression. (a) Immunohistochemical sections. (b) Quantitative graph. Data are expressed as mean ± SEM (*n* = 3) and were analyzed using one‐way ANOVA with Tukey’s test. AETO 85 and AETO 170: aqueous extracts of *Tricalysia okelensis* at doses of 85 and 170 mg/kg, respectively; EETO 85 and EETO 170: ethanolic extracts of *Tricalysia okelensis* at doses of 85 and 170 mg/kg, respectively; Capto 20 = captopril (20 mg/kg).

Figures 9 and 10 show the expression of iNOS in the heart and in the aorta, respectively. It can be noticed that iNOS expression is very weak in the heart of the naive group compared to the aorta. Following exposure to L‐NAME, cardiac iNOS expression rose almost 13 times (*p* < 0.001; *R* ^2^ = 0.89; 95% CI: −13.80 to −4.40), as depicted in Figure 9b. At the level of the heart, treatment of L‐NAME hypertensive rats with captopril, or of *T. okelensis* extracts, significantly reduced iNOS expression (*p* < 0.001, *p* < 0.01, and *p* < 0.05), while the expression of this enzyme was not significantly reduced at the level of the aorta (Figure 10b). The aqueous extract at the dose of 170 mg/kg exhibited the most important decrease in iNOS expression, both at the cardiac and vascular levels (Figures 9b and 10b).

#### 3.7.3. Effect of *Tricalysia okelensis* Leaf Extracts on ANP Expression

**FIGURE 11:**
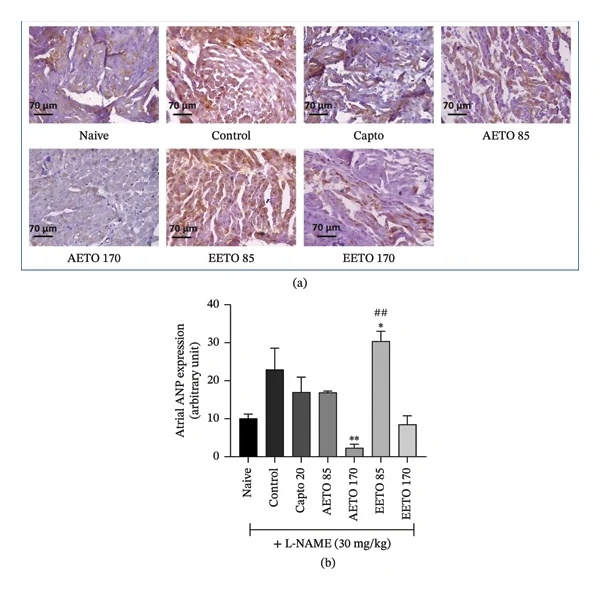
Effects of *Tricalysia okelensis* extracts on atrial ANP expression. (a) Immunohistochemical sections. (b) Quantitative graph. Data are expressed as mean ± SEM (*n* = 3) and were analyzed using one‐way ANOVA with Tukey’s test. ^##^ *p* < 0.01 significantly different compared to the naive. ^∗^ *p* < 0.05, ^∗∗^ *p* < 0.01 significantly different compared to the control group. AETO 85 and AETO 170: aqueous extracts of *Tricalysia okelensis* at doses of 85 and 170 mg/kg, respectively; EETO 85 and EETO 170: ethanolic extracts of *Tricalysia okelensis* at doses of 85 and 170 mg/kg, respectively; Capto 20 = captopril (20 mg/kg).

**FIGURE 12:**
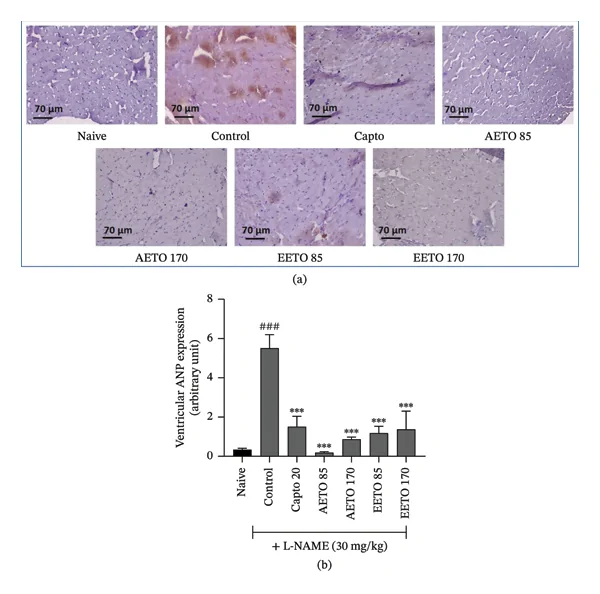
Effects of *Tricalysia okelensis* extracts on ventricular ANP expression. (a) Immunohistochemical sections. (b) Quantitative graph. Data are expressed as mean ± SEM (*n* = 3) and were analyzed using one‐way ANOVA with Tukey’s test. ^###^ *p* < 0.001 significantly different compared to the naive. ^∗∗∗^ *p* < 0.001 significantly different compared to the control group. AETO 85 and AETO 170: aqueous extracts of *Tricalysia okelensis* at doses of 85 and 170 mg/kg, respectively; EETO 85 and EETO 170: ethanolic extracts of *Tricalysia okelensis* at doses of 85 and 170 mg/kg, respectively; Capto 20 = captopril (20 mg/kg).

As depicted in Figures 11 and 12, ANP was more expressed in the atrium than in the ventricle. At the level of the atria, L‐NAME did not significantly (*p* > 0.05; *R* ^2^ = 0.81; 95% CI: −27.31 to 1.57) enhance the expression of ANP (Figure 11), but the ethanolic extract of *T. okelensis* at the dose of 85 mg/kg potentiated the effect of L‐NAME, while the aqueous extract at the dose of 170 mg/kg considerably abated the expression of ANP (Figure 10b). The ventricular ANP expression was almost null in the naive group but significantly increased (*p* < 0.001; *R* ^2^ = 0–84; 95% CI: −7.59 to −2.76) in the control group. This effect was completely abolished when the aqueous extract at the dose of 85 mg/kg was given to animals (Figure 12b). Furthermore, treatment with captopril or ethanolic extract (85 and 170 mg/kg) markedly reduced ventricular ANP expression (*p* < 0.001).

## 4. Discussion

AHT is a medical condition that represents the foremost risk factor for cardiovascular diseases [32]. It is known to promote cardiac and vascular modeling through complex pathophysiological mechanisms [33]. In this study, we investigate the antihypertensive effects of *Tricalysia okelensis* and its ability to reverse L‐NAME‐induced hypertension‐mediated cardiac and vascular remodeling.

L‐NAME is a well‐established and widely used model of experimental hypertension and organ damage within the cardiovascular system that recapitulates the clinical signs of human essential hypertension [12, 34]. In this study, chronic exposure to L‐NAME seriously decreased serum NO and eNOS expression, culminating in an increased systolic and diastolic pressure consistent with the literature. After treatment with *T. okelensis* extracts, NO bioavailability was significantly improved as well as aortic eNOS expression; blood pressure decreased as well. These results clearly show that *T. okelensis* exhibits antihypertensive effects by improving endothelial dysfunction through the stimulation of eNOS expression.

It has been demonstrated that L‐NAME‐induced hypertension is also mediated by the renin‐angiotensin system with Ang II as the main effector [35, 36]. Concordantly, in this study, it was found that L‐NAME administration caused a significant rise in serum Ang II. *T. okelensis* extracts, similar to captopril, an inhibitor of the angiotensin‐converting enzyme, decreased the serum level of Ang II. This result suggests that the modulation of the renin‐angiotensin system (RAS) participates in the antihypertensive effect of *T. okelensis*. Crosstalk has been demonstrated between the NO pathway and the RAS. Indeed, Ding et al. [7] showed that Ang II decreased eNOS phosphorylation, leading to a reduction in eNOS activity, a reduction in NO bioavailability and endothelial dysfunction [8]. The fact that *T. okelensis* but not captopril was able to reverse the reduction in NO clearly indicates that the plant extracts interfere with both the eNOS/NO pathway and the RAS.

The improvement of the eNOS/NO pathway is known to prompt vasorelaxation, which further causes a decrease in blood pressure. We found in a pilot study that *T. okelensis* exhibits vasorelaxant activity. These results suggest the antihypertensive effect of *T. okelensis* may be mediated by an NO‐dependent vasorelaxation activity, though a possible direct action on vascular smooth muscles using other pathways cannot be ruled out.

Sustained elevated blood pressure is a well‐documented and common cause of biological and structural changes at the level of the heart and blood vessels known as remodeling [37, 38]. Cardiac hypertrophy is the typical sign of cardiac remodeling that results from a combination of neurohormonal and mechanical stimuli in response to increased workload [39]. During the process of cardiac remodeling, many structural, molecular, and biochemical changes occur, including enlargement of cardiac cells, cardiomyocyte death, fibrosis, and the reactivation of fetal genes [10, 37]. The findings of this study show that the heart weight to tibia length ratio of animals that received only L‐NAME highly increased, as well as the LV wall to lumen ratio. These results show that L‐NAME induced a concentric cardiac hypertrophy characterized by a reduced LV lumen and an enlarged LV wall [35]. However, although changes in LV wall or lumen in the L‐NAME group were not statistically significant, they were marked enough to be ignored and may indicate an early stage of cardiac hypertrophy. This hypothesis is supported by the elevated ANP expression and the presence of fibrosis as a result of L‐NAME administration, as observed in this study. Rats treated with the ethanolic extract of *T. okelensis* had a significantly low LV wall‐to‐lumen ratio. The reduction in the blood pressure elicited by this plant extract minimized cardiac workload, shielded the heart muscle from hypertension‐mediated heart damage, and therefore, reduced the LV wall‐to‐lumen ratio.

ANP is one of the fetal genes whose reprogramming in the ventricle has been reported to activate pathological cardiac remodeling [40, 41]. ANP is expressed in both atria and ventricles of neonates, but in the adult heart, ventricular expression of ANP declines drastically, while ANP production continues in the atria. Under pathological conditions such as hypertensive cardiac hypertrophy, the ventricular expression of ANP is reactivated in the adult heart [42]. So, targeting these fetal genes could be a good therapeutic option for the management of cardiac hypertrophy. The results of this study show that extracts of *T. okelensis* reversed L‐NAME‐induced ventricular ANP reactivation. This finding is in agreement with the abovementioned antihypertrophic effect of *T. okelensis*. It has also been observed in this study that captopril exhibited an antihypertrophic effect as evidenced by the reduction of the LV wall‐to‐lumen ratio and the inhibition of the ventricular ANP expression. This result clearly demonstrates the role of the renin‐angiotensin system in cardiac remodeling.

ANP and BNP are both markers of cardiac hypertrophy and each of them can be used. In this study, ANP was assessed based on the study by Rubattu et al. [43], which shows that ANP significantly contributes to ventricular remodeling in human essential hypertension compared to BNP. It has been observed in this study that L‐NAME induced a nonsignificant increase in atrial ANP expression, whereas it significantly increased ventricular ANP expression. Indeed, in an adult heart, the major source of ANP comes from the atria under physiological conditions, while ventricular ANP production is very low [42]. Consequently, the atria produce large amounts of ANP. Thus, atrial ANP expression may be less sensitive to further stimulation. An increase in ventricular ANP expression is associated with the reactivation of the fetal gene program following activation of hypertrophic signals triggered by stimuli such as L‐NAME. This may explain why the increase in ANP expression is significant in the ventricle and not in the atrium following L‐NAME administration. The nonsignificant increase in atrial ANP elicited by L‐NAME administration seems to be potentiated by the ethanolic extract of *T. okelensis* at the dose of 85 mg/kg. But this hypothesis is difficult to reconcile with the antihypertensive effect of this extract at the abovementioned dose. This finding requires further validation.

As seen in this study, Ang II levels were increased in rats chronically administered with L‐NAME alone. According to many reports, Ang II plays a central role in L‐NAME‐induced hypertension and organ remodeling [35, 44]. Moreover, Ang II has been shown to activate various pathways leading to cardiac and vascular remodeling, including the increased deposit of collagen fibers known as fibrosis [10]. In this study, a limited sample size (*n* = 3) was used for the histopathological assessment. Although this sample size is small, it meets the minimum requirement to establish proof of concept and complies with the ethical guidelines of the 3Rs. The analysis of histological sections of the heart from the control group (L‐NAME) stained with Masson’s trichrome show areas of cell death that were consistent with the increase in serum LDH and zones of important fibrotic material deposit as evidenced by the increase in CVF. This increase in CVF indicates the development of fibrosis. Treatment with the plant extracts successfully overrides these structural, biochemical, and physiological alterations elicited by L‐NAME, illustrating the cardioprotective effects of *T. okelensis*. Since *T. okelensis* extracts lower serum levels of Ang II, it can be postulated that the cardioprotective effect of this plant arises from an interference with the RAS. In this study, we observed that captopril, an angiotensin‐converting enzyme inhibitor, reversed L‐NAME‐induced hypertension and exhibited an anti‐remodeling effect. This finding supports the role of the renin‐angiotensin system in the development of L‐NAME hypertension and the subsequent cardiac and vascular remodeling. Moreover, the results of this study align with those of Maneesai et al. ^46^ which showed that captopril protects against L‐NAME‐induced cardiac and vascular remodeling.

Although no significant change in the vascular wall was observed among experimental groups, it is worth noticing that L‐NAME caused a thickening of collagen fibers as observed on histological sections. This result suggests that at the vascular level, the process of remodeling was at its initial phase. The thickening of the vascular wall together with reduced expression of eNOS contributes to increased vascular tone and, consequently, elevated blood pressure [45]. Animals that received the plant extracts had arteries with collagen fibers similar in shape to those found in normotensive rats (naive). This result implies that *T. okelensis* impeded vascular remodeling, thereby diminishing vascular tone and lowering blood pressure.

The findings of this study show that after L‐NAME was administered alone, cardiac NO levels were slightly increased rather than decreased as expected. In order to determine the reason behind this result, immunohistological assessment of iNOS expression was undertaken in the heart and in the aorta of all experimental groups. It turns out that cardiac and vascular iNOS expression was increased in the control group (L‐NAME). This result is consistent with that of Leo et al. [46] who found that L‐NAME raises iNOS mRNA expression in the aorta and carotid vessel. This implies that the modest increase in cardiac NO observed in the present work may result from the activity of iNOS. Moreover, this finding clearly shows that inflammation is one of the pathomechanisms responsible for L‐NAME‐induced hypertension and organ damage. Indeed, iNOS has been reported to detrimentally affect the cardiovascular system by promoting eNOS uncoupling through arginase activity upregulation [47]. In addition to iNOS overexpression, MPO levels were increased, and histological sections of the hearts of animals from the control group reveal an infiltration of leukocytes. All these observations are in agreement with an inflammatory process. The administration of the plant extracts downregulates the expression of iNOS, lowers cardiac MPO, and mitigates leukocyte infiltration. These results suggest that *T. okelensis* possesses anti‐inflammatory activity. This anti‐inflammatory effect may also contribute to the anti‐remodeling effect of the plant.

LC‐MS/MS‐based molecular networking analysis supports putative annotation of several polyphenolic metabolites, including chlorogenic acid and neochlorogenic acid, rutin, quercetin derivatives, kaempferol derivatives, isorhamnetin, avicularin, and guaijaverin. Several of these compounds have previously been associated with biological activities consistent with the pharmacological effects observed in the present study. Indeed, they have been reported to exhibit antihypertensive and endothelial protective activities through the modulation of NO/eNOS signaling pathway and attenuation of renin‐angiotensin‐associated cardiovascular dysfunction and anti‐inflammatory mechanisms [36, 48–53]. Rutin has been shown to enhance eNOS activity and NO production while suppressing inflammatory mediators including iNOS [52]. In addition, kaempferol, quercetin, and isorhamnetin derivatives have demonstrated cardioprotective, vasorelaxant, antioxidant, and anti‐remodeling effects in experimental models of cardiovascular dysfunction [36, 48, 49, 53]. Although the biological activity of the extracts cannot be attributed to a single constituent, the presence of these putatively annotated phenolic acids and flavonoids provides a plausible chemical basis for the antihypertensive and anti‐remodeling effects observed for *T. okelensis* extracts in the present study.

## 5. Conclusion

Taken together, this study brings evidence of the potential of *T. okelensis* in the management of hypertension and hypertension‐mediated organ damage. The findings of this study show that *T. okelensis* exhibits antihypertensive effects and greatly reversed hypertension‐induced cardiac and vascular remodeling. Phytochemical characterization of the extracts using LC‐MS/MS‐based molecular networking revealed the presence of several putatively annotated polyphenolic constituents, including chlorogenic acid and neochlorogenic acid, rutin, quercetin derivatives, kaempferol derivatives, isorhamnetin, and guaijaverin, which may collectively contribute to the observed pharmacological effects. These effects may be mediated through the modulation of the renin‐angiotensin system, leading to a decrease in Ang II levels and the stimulation of the NO/eNOS pathway. The inhibition of the renin‐angiotensin system coupled to the stimulation of the NO/eNOS pathway may be responsible for the improvement in cardiac and vascular remodeling observed in animals treated with *T. okelensis* extracts. In addition, the identified phenolic acids and flavonoids have previously been associated with antihypertensive, antioxidant, endothelial protective, and anti‐inflammatory activities, supporting their potential contribution to the biological effects observed in the present study. However, it is worth noticing some limitations of this study: (1) it is not clear if the decrease in ANP and iNOs expression results from a direct action of the plant extract or an indirect effect. (2) LC‐MS/MS‐based molecular networking enabled the putative annotation of several secondary metabolites based on spectral matching and dereplication approaches. However, further isolation and full structural characterization are required to confirm the identity of the bioactive constituents responsible for the observed pharmacological effects.

## Author Contributions

Florence Nokam: conceptualization, data curation, funding acquisition, investigation, formal analysis, methodology, and writing–original draft. Ariane Falone Goumtsa: investigation and formal analysis. Sahar Mofidi Tabatabaei: LC‐MS/MS metabolomics analysis, GNPS2 molecular networking and dereplication, phytochemical annotation, and writing/editing of the metabolomics sections. Cédric Wamba Koho: investigation and formal analysis. Cherif Mouhamed Moustapha Dial: resources and validation. Kevin Joseph Tidgewell: supervision, metabolomics methodology, validation, and writing, review, and editing. Pierre Watcho: project administration, supervision, validation, and writing–review and editing. Elvine Pami Nguelefack‐Mbuyo: conceptualization, data curation, formal analysis, methodology, project administration, visualization, validation, supervision, writing–original draft, and writing–review and editing.

## Funding

This study was supported by the German Academic Exchange Service (DAAD CASE‐G grant no. 57512768).

## Conflicts of Interest

The authors declare no conflicts of interest.

## Supporting Information

Additional supporting information can be found online in the Supporting Information section.

## Supporting information


**Supporting Information** The supporting information contains LC‐MS/MS metabolomics data, molecular networking analyses, dereplication workflows, annotated MS/MS spectra, and pharmacological supporting data for *T. okelensis* extracts. Figure S1 presents the vasorelaxant effect of the aqueous extract, and Figure S2 illustrates histological sections of the aorta stained with hematoxylin and eosin or Masson’s trichrome. Figures S3–S17 and S20 include LC‐MS/MS‐based molecular networking analyses, GNPS2 dereplication workflows, molecular family visualization, and metabolite annotation processes. Figures S18 and S19 present representative LC‐MS traces and annotation of major metabolites including chlorogenic acid derivatives, rutin, and avicularin. Table S1 summarizes total phenolic and flavonoid contents, and Table S2 presents the effects of the extracts on body weight and organ mass in L‐NAME‐induced hypertensive rats.

## Data Availability

Data are available on request from authors.
