# Supplementary material for: The Leaf Aqueous and Ethanolic Extracts of Tricalysia okelensis Hiern. (Rubiaceae) Reverse L‐NAME‐Induced Hypertension, Cardiac, and Vascular Remodeling in Rats
Source: Adv Pharmacol Pharm Sci. 2026 Sep 7;2026:8990646. doi: 10.1155/adpp/8990646 (PMC13548052; doi:10.1155/adpp/8990646)
Supplement: Supplementary file 1 [file ADPP-2026-8990646-s001.docx]

**The leaf aqueous and ethanolic extracts of *Tricalysia okelensis* Hiern. (Rubiaceae) reverse L-NAME-induced hypertension, cardiac, and vascular remodeling in rats through the inhibition of Ang II, ventricular ANP, iNOS, and upregulation of eNOS**

Florence Nokam^1^, Ariane Falone Goumtsa^1^, Sahar Mofidi Tabatabaei^2^, Cédric Wamba Koho^1^, Cherif Mouhamed Moustapha Dial^3^, Kevin Joseph Tidgewell^2^, Pierre Watcho^1^, Elvine Pami Nguelefack-Mbuyo^1*^

*^1^Research Unit of Animal Physiology and Phytopharmacology, Department of Animal Biology, Faculty of Science, University of Dschang, P.O. Box 67 Dschang, Cameroon*

*^2^Department of Pharmaceutical Sciences, University of Kentucky, Lexington, KY 40536, United States*

*^3^Cheikh Anta Diop University, Faculty of Medicine, Pharmacy and Odonto-stomatology, Dakar, Senegal.*

**Authors’ details**

Florence Nokam: [florencenokam@yahoo.fr](mailto:florencenokam@yahoo.fr); ORCID: 0000-0003-1896-7570

Ariane Falone Goumtsa: [arianfgoumtsa@yahoo.fr](mailto:arianfgoumtsa@yahoo.fr); ORCID: 0000 0003 4789 3750

Sahar Mofidi Tabatabaei: sahar.mofidi@uky.edu, ORCID: 0000-0002-6193-8137

Cédric Wamba Koho: [kohowamba.cedric@gmail.com](mailto:kohowamba.cedric@gmail.com); ORCID: 0009-0003-1634-0624

Cherif Mouhamed Moustapha Dial: [dialcherif@yahoo.fr](mailto:dialcherif@yahoo.fr); ORCID: 0000-0002-3068-8892

Kevin Joseph Tidgewell: Kevin.Tidgewell@uky.edu, ORCID: 0000-0002-0501-2604

Pierre Watcho: [pwatcho@yahoo.fr](mailto:pwatcho@yahoo.fr); ORCID: 0000-0001-5022-3091

Elvine Pami Nguelefack-Mbuyo: [elvine.mbuyo@univ-dschang.org](mailto:elvine.mbuyo@univ-dschang.org); ORCID: 0000 0002 5754 7686

***Corresponding author**

Elvine Pami Nguelefack-Mbuyo, Research Unit of Animal Physiology and Phytopharmacology, Department of Animal Biology, Faculty of Science, University of Dschang, P.O. Box 67 Dschang, Cameroon. Tel.: (+237) 699 37 97 81.

Email address: [elvine.mbuyo@univ-dschang.org](mailto:elvine.mbuyo@univ-dschang.org/mbuyopamielvine@gmail.com)

FIGURE S1**:** Vasorelaxant effect of the aqueous extract of *Tricalysia okelensis* (AETO) on intact aorta rings precontracted with norepinephrine. Each point represents the mean ± SEM of six different experiments. AETO = aqueous extract of *Tricalysia okelensis.*

**Table S1:** Total phenols and flavonoids amount of the aqueous and ethanolic extracts from *T. okelensis* leaves.

|  | **Total phenols**  **(mg GAE/g extract)** | **Total flavonoids**  **(mg QE/g extract)** |
| --- | --- | --- |
| **AETO** | 181.50 ± 7.80 | 785.70 ± 54.08 |
| **EETO** | 138.10 ± 3.17^***^ | 414.30 ± 101.40^**^ |

Experiments were performed in triplicated. Data are expressed as mean ± SEM. ^**^p < 0.01; ^***^p < 0.001 significant difference compared to aqueous extract. GAE = gallic acid equivalent; QE = quercetin equivalent. AETO = aqueous extract of *Tricalysia* and EETO = ethanolic extract of *Tricalysia*

**Table S2:** Effects of the aqueous and ethanolic extracts from the leaves of *Tricalysia okelensis* on the bodyweight and organ mass of rats rendered hypertensive following chronic L-NAME administration.

| **Groups** | **Bodyweight gain (W6-W0)**  **(g)** | **Heart weight/tibia length (mg/cm)** | **Left ventricle weight/tibia length (mg/cm)** | **Aorta weight/tibia length (mg/cm)** |
| --- | --- | --- | --- | --- |
| **Naive** | 49.44 ± 6.37 | 213.40 ± 8.02 | 139.90 ± 6.41 | 20.52 ± 1.66 |
| **Control** | 35.22 ± 3.32 | 257.10 ± 7.73^###^ | 164.30 ± 6.21 | 23.17 ± 2.79 |
| **Capto 20** | 27.88 ± 3.84 | 220.80 ± 5.93^**^ | 134.00 ± 8.27^*^ | 22.13 ± 2.55 |
| **AETO 85** | 40.78 ± 10.71 | 215.90 ± 6.48^***^ | 151.70 ± 5.74 | 18.52 ± 2.17 |
| **AETO 170** | 9.78 ± 11.57^#^ | 205.60 ± 3.57^***^ | 130.00 ± 3.81^**^ | 19.47 ± 2.24 |
| **EETO 85** | 32.44 ± 6.38 | 198.50 ± 3.93^***^ | 146.70 ± 4.08 | 16.24 ± 0.97 |
| **EETO 170** | 15.67 ± 8.25 | 214.10 ± 7.20^***^ | 140.90 ± 6.38 | 13.54 ± 1.01^*^ |

Data are expressed as mean ± SEM (n = 8-9). Data were analyzed using ANOVA one-way with Tukey’s test. ^#^p < 0.05, ^###^p < 0.001 significantly different compared to the naive group; ^**^p < 0.01 ^***^p < 0.001 significantly different compared to the control group ; Capto 20 = captopril (20 mg/kg); AETO 85 and AETO 170 = aqueous extract of *Tricalysia okelensis* at the doses of 85 and 170 mg/kg, respectively; and EETO 85 and EETO 170 = ethanolic extract of *Tricalysia okelensis* at the doses of 85 and 170 mg/kg, respectively

**a**

**b**

FIGURE S2**:** Effects of *Tricalysia okelensis* on histological sections of the aorta stained with hematoxylin and eosin (a) or Masson’s trichrome (b). Magnification X400. Panels (c) and (d) depicted the histomorphometric analysis. Data are expressed as mean ± SEM (n = 3) and were analyzed using ANOVA one-way with Tukey post test. Capto 20 = captopril (20 mg/kg); AETO 85 and AETO 170: aqueous extract of *T. okelensis* at the doses of 85 and 170, respectively; EETO 85 and EETO 170: ethanolic extract of *T. okelensis* at the doses of 85 and 170 mg/kg, respectively. A: adventitia; M: media; E: endothelium.

FIGURE S3: Molecular Networking analysis of *Tricalysia okelensis* aqueous extract using LC-MS/MS (positive mode)

FIGURE S4: Extracted ion chromatogram and MS/MS spectral comparison (experimental vs. library) for Spiraeoside

FIGURE S5: Extracted ion chromatogram and MS/MS spectral comparison (experimental vs. library) for Quercetin

FIGURE S6: Extracted ion chromatogram and MS/MS spectral comparison (experimental vs. library) for Neochlogeneric acid

FIGURE S7: Extracted ion chromatogram and MS/MS spectral comparison (experimental vs. library) for Rutin

FIGURE S8: Extracted ion chromatogram and MS/MS spectral comparison (experimental vs. library) for Kaempferol|3,5,7-trihydroxy-2-(4-hydroxyphenyl)chromen-4-one

FIGURE S9: Extracted ion chromatogram and MS/MS spectral comparison (experimental vs. library) for quercetin-3-O-pentoside

FIGURE S10: Molecular Networking analysis of *Tricalysia okelensis* ethanolic extract using LC-MS/MS (positive mode)

FIGURE S11: Extracted ion chromatogram and MS/MS spectral comparison (experimental vs. library) for Chlorogenic Acid

FIGURE S12: Extracted ion chromatogram and MS/MS spectral comparison (experimental vs. library) for Kaempferol-7-neohesperidoside

FIGURE S13: Extracted ion chromatogram and MS/MS spectral comparison (experimental vs. library) for Kaempferol|3,5,7-trihydroxy-2-(4-hydroxyphenyl)chromen-4-one

FIGURE S14: Extracted ion chromatogram and MS/MS spectral comparison (experimental vs. library) for kaempferol 3-O-beta-D-xyloside

FIGURE S15: Extracted ion chromatogram and MS/MS spectral comparison (experimental vs. library) for Spiraeoside

FIGURE S16: Extracted ion chromatogram and MS/MS spectral comparison (experimental vs. library) for Rutin

FIGURE S17**:** Extracted ion chromatogram and MS/MS spectral comparison (experimental vs. library) for Isorhamnetin

FIGURE S18: LC-MS of the aqueous extract showing the major compounds

FIGURE S19: LC-MS of the ethanolic extract showing the major compounds

FIGURE S20: Extracted ion chromatogram and MS/MS spectral comparison (experimental vs. library) for Guaijaverin
